# Specific Rhizobacteria Responsible in the Rhizosheath System of *Kengyilia hirsuta*

**DOI:** 10.3389/fpls.2021.785971

**Published:** 2022-01-28

**Authors:** Youjun Chen, Chen Chen, Qingping Zhou, Jian Hu, Yingxia Lei, Wenhui Liu

**Affiliations:** ^1^Sichuan Zoige Alpine Wetland Ecosystem National Observation and Research Station, Chengdu, China; ^2^Institute of the Qinghai-Tibetan Plateau, Southwest Minzu University, Chengdu, China; ^3^Qinghai Academy of Animal Science and Veterinary Medicine Qinghai University, Xining, China

**Keywords:** *Kengyilia hirsuta*, rhizobacteria, rhizosheath, formation, aggregation

## Abstract

The rhizosheath is a critical interface supporting the exchange of resources between plants and their associated environment of soil. Favorable microenvironment of rhizosphere soil provides the rhizosheath formed and then promotes desert plant survival. However, it remains unclear how rhizosheath benefits the colonization of pioneer plants in alpine desert under changing environment. In this study, we investigated the effect of different soil moisture and sterilization treatments (three moisture levels and unsterilized or sterilized soil) on rhizosheath forming process of *Kengyilia hirsuta* (*K. hirsuta*), a sand-inhabiting and drought-resistant pioneer plant of the Tibetan Plateau desert. The results showed that in both unsterilized and sterilized soil, increasing soil moisture first increased and then decreased rhizosheath weight, with the highest value is 25%. During rhizosheath formation, developing rhizosheaths were selectively enriched in the bacterial genera *Massilia* and *Arthrobacter*. These suggest the existence of a highly specialized signal recognition system during rhizosheath formation that involves the accumulation of bacteria. These bacterial species exhibited different roles in the process of rhizosheath formation and is an advantageous strategy for *K. hirsuta*.

## Introduction

The genus *Kengyilia* (Triticeae) comprises a group of pioneer plant species predominantly distributed on sandy grasslands of the Tibetan Plateau (TP) and adjacent northern areas in China. The phylogenetically most advanced species, *Kengyilia hirsuta* (*K. hirsuta*), is found at the highest elevations of the TP region (Baum et al., 1995). The composition of the sandy grassland plant community in sand grassland is driven by sandy desertification and is related to plant functional traits in the presence of environmental changes (Yue, 2017). As a pioneer species of the sandy desert plant community and a well-adapted forage grass, *K. hirsuta* is able to accommodate different environmental stresses.

The rhizosheath is defined as the portion of soil that physically adheres to the root system and encases the entire root system of certain plants (Baum et al., 1995; Pang et al., 2017). Plant root hairs, soil moisture (Pang et al., 2017), fungal hyphae of soil microbes, and microbial- and plant-derived mucilage are responsible for the aggregation of sand particles in the rhizosheath system (Marasco et al., 2018). The beneficial effect of the rhizosheath has been demonstrated by the observation of a positive correlation between rhizosheath mass and plant growth under stress conditions (Ashraf et al., 2006). In grasslands undergoing desertification, the rhizosheath has been shown to provide mechanical protection to roots, thereby promoting water conservation and uptake under drought conditions (Young, 2006; Brown et al., 2017). In addition, the rhizosheath can serve as a resource for microorganisms, which can feed on organic substances and live in relatively stable environmental conditions compared with barren sandy land. Meanwhile, the rhizosheath structure provides an ecological niche with available microclimatic conditions for microbial growth and development (Danin, 1996). A rhizosheath-root system is formed around the roots of grasses in families Poaceae and Haemodoraceae (Young, 2006; Brown et al., 2017); coincidentally, we observed the rhizosheath in *K. hirsuta* root. The ability of *K. hirsuta* to inhabit arid environments may primarily be related to the presence of a rhizosheath. As it is well known, moisture and bacteria are limited factors for desert plant survival and growth.

According to a report, a comparison of rhizosheaths in different soil layers revealed that rhizosheaths in the dry layer were five times the volume of the subtending root, whereas rhizosheath volumes were only 1.5 times larger than those of the root in the wet layer in field (Brown et al., 2017). In the dry layer, the mucilage of roots and microorganisms enhanced their adhesiveness for the stabilization of the rhizosheath; as a consequence, the rhizosheath was more easily removed from roots growing in wet soil than dry soil (Watt et al., 1994). In the process of rhizosheath formation, the microbial community plays an important role (Pointing and Belnap, 2012). Soil microbes stimulate plant roots to produce more mucilage, which promotes rhizosheath formation; besides, soil microbes decompose and use soil organic matter for rhizosheath formation (Marasco et al., 2018). As we known that a few microbial cultivation-based studies have been conducted on the root system of desert grasses (Othman et al., 2004; Hanna et al., 2013), for example, two phyla (Glomeromycota and Zygomycota) and two trophic modes (Symbiotroph and Pathotroph-Saprotroph) were the differentially ecological functions in monitoring desertification conditions, they can be used to indicate desertification gradient (Zong and Fu, 2021).

Soil sterilization may alter the root growth and community structure of newly developed bacterial populations (Wertz et al., 2007; Marasco et al., 2012) and, thus, also influence rhizosheath development.

Taken together, soil moisture and the specific microorganisms that strongly influence plant rhizosheaths (Watt et al., 1994; Danin, 1996), but the information on the development of rhizosheaths has been lacking. Given these issues, we studied *K. hirsuta* rhizosheath weight in unsterilized and sterilized desert soil at three different moisture levels. Next, high-throughput sequencing technology was used to explore whether specific rhizobacteria are responsible for the aggregation of sand particles in the rhizosheath system of *K. hirsuta*. Finally, experiments were conducted to examine their possible role in altering the root growth and the weight of rhizosheaths. The key point of this study was to discuss the special rhizobacteria that could help *K. hirsuta* to better developing rhizosheaths in different moisture and environment of soil.

## Materials and Methods

### Sampling Approach

#### Seed Collection

In September 2017, seeds of healthy, same-sized seedlings of *K. hirsuta* growing in northwest of Sichuan, China (elevation, 3,450 m; longitude, N 33°18′, latitude, E 102°62′) were selected for cultivation.

#### Soil Samples

The soil used in laboratory experiments was collected from an experimental field at the Institute of the TP, Southwest Minzu University, Hongyuan, China. The field, which had been enclosed for the past 10 years, contained plots harboring mainly shrub and grass species such as *Hippophae rhamnoides*, *Orobanche coerulescens*, *Leymus secalinus*, *Artemisia desertorum*, *Potentilla anserine*, and *Kobresia pygmaea*.

Soil sterilization may alter the root growth and community structure of the bacterial diversity and abundance (Marschner and Bredow, 2002; Wertz et al., 2007), So, we choose the autoclaving method that may also influence the development of rhizosheaths. Autoclaving is known to alter physicochemical characteristics of soil (Mcnamara et al., 2003; Berns et al., 2010; Traoré et al., 2010). Autoclaving is known to increase the soil pH (Jörn et al., 1993), to decrease the cation exchange capacity (CEC), and increasing the electrical conductivity (EC) of soil and N (NH_4_^+^ and NO_3_^–^) is not an significant change (Sandler et al., 1988). After collection, the sampled soil was divided into two portions, one of which was sterilized by autoclaving at 121°C for 1 h. Soil physicochemical characteristics are given in Table 1.

**TABLE 1:** Physicochemical properties of sandy soil used in this study.

Parameter	Treatment of soil	
	Sterilization	Unsterilization
Field capacity(%)	21.31 ± 2.01a	20.58 ± 1.34a
pH (1:1,soil:water)	7.68 ± 0.02a	7.23 ± 0.01b
Electrical conductivity (μ⋅S⋅m^–1^)	531.10 ± 7.45a	349.75 ± 3.12b
NH4 + N (mg⋅kg^–1^)	9.82 ± 1.11a	9.44 ± 1.28a
NO3−N (mg⋅kg^–1^)	4.61 ± 0.73a	3.80 ± 0.52a

*Different lowercase letters in the same row indicate significant differences (p < 0.05).*

### Plant Growth Experiments

Experiments were conducted in plastic flower plots (5.5 cm inner diameter × 7 cm depth) filled with 750 g of sterilized or unsterilized soil. Nine seeds of *K. hirsuta* were sown per pot. The final soil moisture content of each pot was adjusted to 10, 25, or 40% of field water capacity and maintained throughout the experiment as indicated by daily weighing. All the experiments were repeated four times per treatment in an artificial climate incubator in October. Experimental conditions were as follows: 14-h/10-h light-dark photoperiod, 25°C, 55% relative humidity, and 1,000–1,200 μmol m^–2^ s^–1^ photon flux density. To study the effects of moisture and soil treatments on plant growth, we investigated the rhizosheath soil of seedlings at the three-leaf stage. In the first experiment, we analyzed the weight of rhizosheath soil for insoluble saccharides (Delhaize et al., 2012). Differences between treatments of rhizosheath weight were tested with the two-way ANOVA. In the second experiment, we examined the microbial diversity of the rhizosheath soil.

### Deoxyribonucleic Acid Extraction and Sequencing

Total DNA was extracted from 0.5 ± 0.05 g samples of sandy rhizosheath soil subjected to different moisture and sterilization treatments using the DNA Isolation Kit (BioTeke Corporation, Beijing, China). The bacterial 16S ribosomal RNA (16S rRNA) gene was PCR amplified in triplicate from the extracted DNA using primers 27 F and 1,492 R (Bolger et al., 2014). For high-throughput sequencing, each rhizosphere soil sample was extracted using the Power Soil DNA Isolation Kit. DNA quality was monitored by 0.8% agarose gel electrophoresis and the extracted DNA was diluted to a concentration of 1 ng/μl and stored at –20°C until further processing. The diluted DNA was used as a template for PCR amplification of bacterial 16S rRNA genes with bar-coded primers and the HiFi HotStart ReadyMix (Kapa).

For the bacterial diversity analysis, V3-V4 variable regions of 16S rRNA genes were amplified with universal primers 343 F and 798 R (Tanja and Salzberg, 2011). Amplicon quality was checked by gel electrophoresis. The amplified DNA was purified with AMPure XP beads (Agencourt Bioscience Corporation, Shanghai, China) and subjected to another PCR amplification round. After repurification with AMPure XP beads, the final product was quantified using the Qubit dsDNA Assay Kit. Equal amounts of purified DNA were pooled for subsequent sequencing at Shanghai OE Biotechnology Corporation (Shanghai, China) using the MiSeq platform.

### Processing of High-Throughput Sequencing Data

High-throughput sequencing yielded raw sequencing data in FASTQ format. The paired-end reads were preprocessed using Trimmomatic software version 0.36 (Caporaso et al., 2010) to detect and remove ambiguous (“N”) bases. We then removed low-quality sequences with an average quality score below 20 using a sliding window trimming approach. After trimming, we used fast length adjustment of short read (FLASH) software version 1.2.11 to assemble paired-end reads (Edgar et al., 2011). Parameters of the assembly were 10-bp minimal overlap, 200-bp maximum overlap, and 20% maximum mismatch rate. To remove additional noise from the data, reads with ambiguous, homologous sequences and those containing fewer than 200 bp were eliminated, while reads with 75% of bases above Q20 were retained. Finally, chimeric reads were detected and removed. These two steps were achieved using quantitative insights into microbial ecology (QIIME) software version 1.8.0 (Rognes et al., 2016). Clean reads were subjected to primer sequence removal and clustered to generate operational taxonomic units (OTUs) using UPARSE software with a 97% similarity cutoff (Amann et al., 1990). Representative reads for each OTU were selected using QIIME software. All the representative reads were annotated and blasted against the Silva database (version 123) using ribosomal database project (RDP) classifier with a confidence threshold of 70% (Subbureddiar et al., 2007).

### Analysis of High-Throughput Sequencing Data

Alpha diversity was calculated using the R version 3.4.3 “vegan” package and plotted using Origin 2017. The Mann–Whitney *U* test was used to test differences in alpha diversity between groups. A heatmap of the top 30 genera was generated with the R “pheatmap” package. Non-metric multidimensional scaling (NMDS) based on Morisita–Horn and Jaccard dissimilarities was performed using the R “vegan” package to investigate differences among rhizosheath bacterial communities in the different treatment samples. Permutational multivariate ANOVA was used to test for statistically significant differences among moisture degree or soil sterilization treatment on the basis of bacterial OTU richness or bacterial phylogenetic diversity (0/1 matrix) and was performed using the “vegan” R package. Venn diagrams were generated using the R “VennDiagram” package. All other graphs were generated in Origin 2017.

### Growth-Promoting Activity of Bacterial Operational Taxonomic Units for *Kengyilia hirsuta*

Three bacterial strains were chosen for soil inoculation experiments: OTU16 (*Arthrobacter* strain A6; GenBank accession number NC_011886), OTU6 (*Sphingomonas* strain S-130; NZ_BBWU01000045), and OTU10 (*Massilia* strain CP177-4; KY945685). Plump seeds of *K. hirsuta* collected from northwestern Sichuan in China were selected for experiment. The seeds were surface disinfected in 75% ethanol for 3 min followed by 0.5% NaClO for 3 min, rinsed in sterile water, and placed in a sterile plastic cup (7 cm diameter × 7.5 cm height) with 650 g of sterilized soil. The bacteria were incubated in tryptic soy broth for 24 h and then diluted to an OD_600_ of 0.5–0.6 using a spectrophotometer (Thermo Fisher Scientific, Chengdu, China). Bacterial suspensions (3 ml) were added to the soil of 7-day-old seedlings, with sterilized bacterial suspension used as a control. Each plant–bacterial OTU combination included four replicates. The plants were grown for 60 days (25°C, 55% humidity, 14-h/10-h day/night photoperiod, 1,000–1,200 μmol m^–2^ s^–1^ illumination intensity) until harvested to measure root and rhizosheath weights. Sterilized water was used to maintain moisture as needed. Root and rhizosheath weights were plotted using Origin 2019 and differences between treatments and controls were tested with the two-way ANOVA, the Mann–Whitney *U* test, and the Kruskal–Wallis test using the SPSS software version 22 (Chengdu, China).

## Results

### *Kengyilia hirsuta* Rhizosheath Under Different Water and Soil Sterilization Treatment Conditions

**FIGURE 1:**
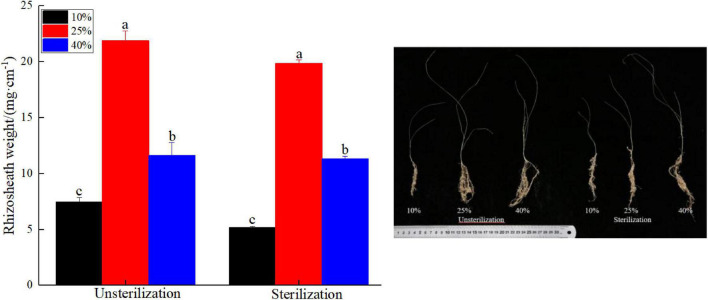
The differences in rhizosheath weight due to different soil and moisture treatment. Different lowercase letters in the figure indicate significant differences (*p* < 0.05).

Rhizosheaths were found in all the plant samples. Under different soil moisture treatments, rhizosheath weights varied from 7.46 to 21.90 mg cm^–1^ in unsterilized soil and from 5.16 to 19.88 mg cm^–1^ in sterilized soil (Figure 1). The interaction between soil treatment and water difference in rhizosheath weight was not significant (Supplementary Figure 1, *p* > 0.05). Rhizosheath weights observed at the 25% moisture level in unsterilized and sterilized soil conditions 21.90 and 19.88 mg cm^–1^, respectively, were significantly higher (*p* < 0.05) than the corresponding weights obtained at the two other moisture levels.

### Profile of Bacteria Based on High-Throughput Sequencing

Sequencing of 18 sterilized and 18 unsterilized rhizosphere soils generated 1,312,862 valid raw reads. A total of 20,783 to 44,698 sequencing reads, of which 80.21 to 86.51% were valid, were generated per sample (Supplementary Table 1). After filtering out OTUs associated with mitochondria and chloroplasts, an OTU table was generated for further analysis. The rarefaction curve reached saturation at approximately 12,000 sequences and the number of species was higher in unsterilized than sterilized soil (Supplementary Figure 2).

### Bacterial Diversity Based on Alpha and Beta Indexes

To investigate the bacterial component and diversity in informed processing of *K. hirsuta* rhizosheath, we compared the bacterial diversity of the treatment groups using different alpha indexes. According to the Chao index, the abundance of rhizosphere soils in the sterilized and unsterilized treatment groups was significantly different (Mann–Whitney *U* test, *p* < 0.05). Identically, a comparison revealed significant differences among moisture levels (Supplementary Table 2). Bacterial diversity was also measured using the Shannon and Simpson indexes (Supplementary Figure 3). According to Simpson index, rhizosheaths in unsterilized soil were more diverse than those in sterilized soil (*p* < 0.05) and similar were found among moisture levels in both the unsterilized and sterilized soil treatment groups (*p* > 0.05).

A total of 27 phyla and 16 classes were identified by high-throughput sequencing (Supplementary Table 3). Proteobacteria (41.12%), Actinobacteria (28.87%), Gemmatimonadetes (12.87%), and Bacteroidetes (9.44%) were the dominant phyla. The most abundant classes were Actinobacteria (20.28%), Betaproteobacteria (17.70%), Alphaproteobacteria (15.64%), Gemmatimonadetes (12.87%), Sphingobacteriia (6.98%), Thermoleophilia (5.43%), Gammaproteobacteria (4.09%), Deltaproteobacteria (3.56%), Acidobacteria (2.46%), Acidimicrobiia (2.38%), Cytophagia (2.34%), and Bacilli (2.21%). The following genera and generic categories accounted for 65.75% of total reads: other (24.46%), uncultured (12.31%), *Gemmatimonas* (5.97%), *Sphingomonas* (5.30%), *Arthrobacter* (5.14%), *Pseudonocardia* (4.00%), uncultured bacterium (3.65%), *Patulibacter* (2.75%), and ambiguous taxa (2.17%).

### Enrichment of Specific Soil Bacteria in the *Kengyilia hirsuta* Rhizosheath Under Sterilization and Water Treatments

**FIGURE 2:**
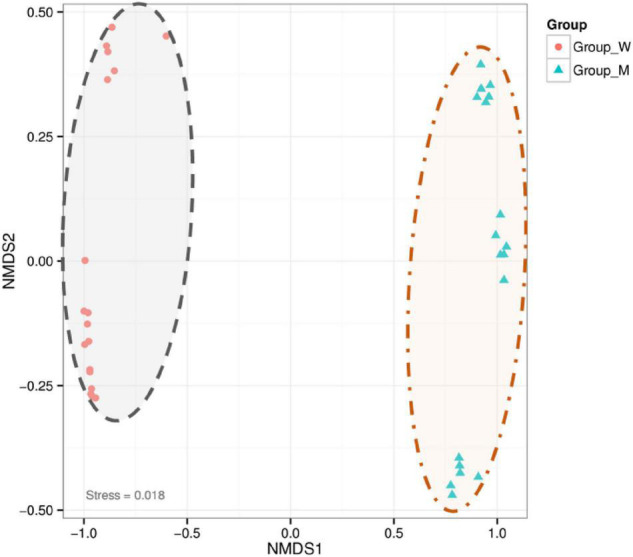
Non-metric multidimensional scaling (NMDS) comparison of bacterial communities in the different treatment groups. The group W represents unsterilized treatment sample in graph and the group M represents sterilized treatment sample in graph.

Because our experiments indicated that rhizosheaths formed under all the tested conditions and that rhizosheath weights varied among soil sterilization and moisture level treatments, the specific bacteria were enriched in developing *K. hirsuta* rhizosheaths in response to particular treatment conditions. The NMDS analysis clearly divided the bacterial community into two groups on the basis of treatment in abscissa (Figure 2), namely, soil treatment (unsterilized and sterilized soil groups) and water treatment (10, 25, and 40%) (Jaccard dissimilarity index, *p* = 0.001). According to these results, the amount of variation due to differences in moisture levels was smaller than that arising from the soil sterilization treatment. Significant differences were observed in the bacterial community between the soil treatment groups (sterilized and unsterilized soil) and among moisture levels (10, 25, and 40%). According to the NMDS analysis, the soil sterilization treatment was responsible for the largest percentage of explained variation in beta diversity.

**FIGURE 3:**
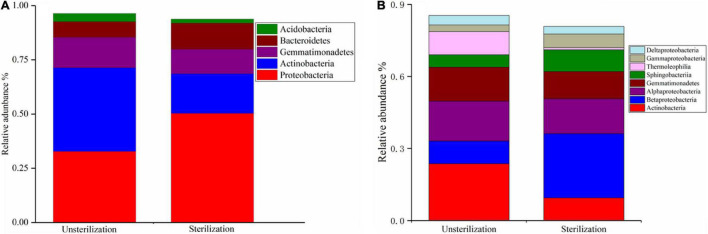
Relative abundance of bacterial phyla under different soil sterilization treatments **(A)**. Relative abundance of bacterial classes under different soil sterilization treatments **(B)**.

We first compared the bacterial community in soil sterilization treatment groups (unsterilized vs. sterilized) at different phylogenetic levels. At the phylum level, the relative abundance of Actinobacteria was significantly higher (*p* < 0.01) in rhizosheaths in unsterilized soil (38.49%) than in sterilized soil (18.22%). Proteobacteria and Bacteroidetes were significantly less represented (*p* < 0.01) in the unsterilized soil group (32.81 and 7.21%, respectively) than in the sterilized soil group (50.32 and 12.01%, respectively). Gemmatimonadetes was common in both the unsterilized (14.10%) and sterilized (11.37%) groups (Figure 3A).

At the class level, Actinobacteria dominated in the unsterilized soil group, representing 23.67% of OTUs in unsterilized conditions compared with 16.37% in rhizosheaths in sterilized soil (*p* < 0.01). The relative abundance of Betaproteobacteria was significantly higher (*p* < 0.01) in the sterilized group (26.74%) than in the unsterilized group (9.44%). Members of Alphaproteobacteria were more abundant in rhizosheaths in unsterilized soil (16.60%) than in sterilized soil (14.50%); a similar result was observed for Gemmatimonadetes: 14.10% in unsterilized soil vs. 11.37% in sterilized soil (Figure 3B).

**FIGURE 4:**
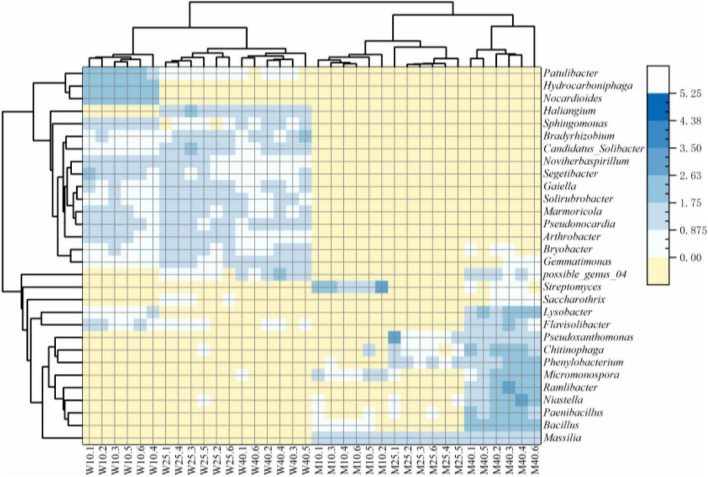
Heatmap of relative abundances of the top 30 genera from the different treatment.

As shown in the heatmap (Figure 4), bacterial communities were divided at the genus level into two major groups on the basis of soil sterilization and moisture treatment. *Sphingomonas*, *Arthrobacter*, *Pseudonocardia*, *Patulibacter*, *Haliangium*, *Solirubrobacter*, *Noviherbaspirillum*, *Marmoricola*, *Candidatus-Solibacter*, *Gaiella*, and *Bradyrhizobium* were significantly more abundant in the unsterilized than sterilized groups (*p* < 0.05, Mann–Whitney *U* test). In contrast, the abundances of *Massilia*, *Flavisolibacter*, *Lysobacter*, *Bacillus*, *Niastella*, *Phenylobacterium*, *Ramlibacter*, *Micromonospora*, *Streptomyces*, *Saccharothrix*, *Chitinophaga*, *Pseudoxanthomonas*, and *Paenibacillus* were significantly higher in the sterilized group than the unsterilized group (*p* < 0.05, Mann–Whitney *U* test). Other genera, such as *Gemmatimonas*, *Nocardioides*, *Bryobacter*, *Segetibacter*, *possible_genus_04*, and *Hydrocarboniphaga* were equally represent in rhizosheaths developed in unsterilized and sterilized soils.

**FIGURE 5:**
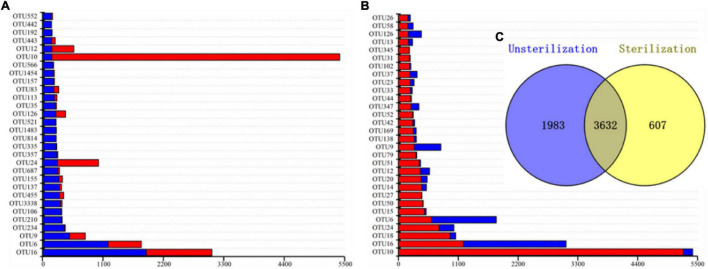
Asymmetrical distribution of operational taxonomic units (OTUs) in the unsterilization soils and sterilization soils. Cumulative histogram of the top 30 bacterial OTUs isolated from *Kengyilia hirsuta* rhizosheaths in unsterilized soil **(A)**. Cumulative histogram of the top 30 bacterial OTUs isolated from *Kengyilia hirsuta* rhizosheaths in sterilized soil **(B)**. Number of OTUs present under different soil sterilization treatment conditions **(C)**. The relative abundance of each OTU, depicted in red (from sterilized soil) or blue (from sterilized soil), is expressed as the log2(x + 1)-transformed number of sequencing reads from a given isolation source.

In total, 3,632 OTUs were present in both the unsterilized and sterilized soil groups, whereas 1,983 OTUs were present only in rhizosheaths from unsterilized soil and 607 OTUs were unique to sterilized soil conditions (Figure 5C). More than 86% of OTUs in the sterilized soil group were also found in the unsterilized group, thus indicating a high overlap between the two bacterial communities. Next, we compared the distribution of the top 30 bacterial OTUs between the sterilized and unsterilized soil treatment groups (Figures 5A,B). We found that OTU16, OTU9, OTU10, and OTU6 were abundant in rhizosheaths from both types of soil.

Among the top 30 OTUs, OTU16 (18.56%) and OTU6 (11.72%) were the most dominant OTUs in the unsterilized soil group. In contrast, OTU10 was predominant in the sterilized soil treatment group. Bacteria in the rhizosheath were, thus, most likely selectively recruited from soil. The five most abundant OTUs in the rhizosheath that were common to both soil types were OTU16 (*Arthrobacter*), OTU6 (*Sphingomonas*), OTU126 (*Gemmatimonas*), OTU10 (*Massilia*), and OTU12 (*Sphingomonas*). Most OTUs belonged to genera *Massilia* and *Arthrobacter* accounted for over 60% of reads. The bacteria in developing rhizosheath in the two soil treatment groups were, thus, different.

### Plant Growth Promotion of Bacteria Isolated From *K. hirsuta* Rhizosheath

**FIGURE 6:**
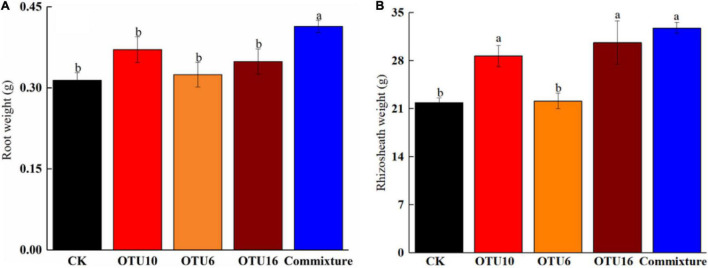
Root-weight-promoting effects of bacterial strains isolated from *K. hirsuta* rhizosheaths **(A)**. Rhizosheath weight-promoting effects of bacterial strains isolated from *K. hirsuta* rhizosheaths **(B)**. The black column represent no infection (CK), the red column represent with OTU10 infected, the yellow column represent with OTU6 infected, the brown column represent with OTU16 infected, the blue column represent with commixture infected (OTU10, OTU6, and OTU16).

Three bacterial strains, OTU16, OTU6, and OTU10, were used in this experiment. Weights of *K. hirsuta* roots grown in sterilized soil inoculated with OTU6, OTU10, or OTU16 were not significantly different (*p* > 0.05) from those of the control, but root weights were significantly increased (*p* < 0.05) by inoculation of soil with a mixture of three strains (Figure 6A). Although inoculation of sterilized soil with OTU6 had no significant effect on the rhizosheath weight of *K. hirsuta* (*p* > 0.05), rhizosheath weights were significantly increased (*p* < 0.05) by inoculation with OTU16, OTU10, and a mixture of three strains (Figure 6B).

## Discussion

### Rhizosheath Weight Changes in Soil Moisture and Sterilization

*Kengyilia hirsuta* has an advanced rhizosheath structure that aids the successful establishment of this species in sand (Oláh, 2004). The rhizosheath structure comprises soil particle and water from soil, the root architecture, root exudates, and soil microorganisms (Young, 2006). Both treatments of unsterilized and sterilized soils, we observed the effect of soil moisture level (10, 25, and 40%) on rhizosheath weight and *K. hirsuta* plants with their robust rhizosheaths were more successfully established in both unsterilized and sterilized soil at the 25% moisture level. Similar results have produced in some earlier studies; the rhizosheath volume in the dry soil layer was five times that of the subtending root, but only 1.5 times the volume of the root in the wet layer. Rhizosheaths are thicker and held to the root with greater tenacity when formed in the drier soils of midsummer and they are less substantial and more easily removed from roots growing in the wetter soils of early spring; when the root is growing under relatively dry conditions, the mucilage is a crucial element for soil adhesion (Watt et al., 1994). Therefore, rhizosheaths easily formed in relatively drier soil.

Our data indicated that soil sterilization reduced the EC and pH of soil, but the rhizosheath weight was not affected; such changes was observed in this study too (Lynch, 1982). This is because in sterilized soil, it probably favored the growth of efficient exopolysaccharide (EPS)-producing bacteria, thus causing increased soil aggregation around roots and the root exudate, e.g., phytohormones, plays a central role in the recruitment of bacteria in rhizosheath informed processes (Mahmood et al., 2014). In this study, the result showed that special bacteria can be recruited in rhizosheath formed processes.

### Bolting the Special Bacteria in Rhizosheath Formed Processes

The structure of the rhizosheath generates a favorable bacteria for the establishment of complex diverse bacterial populations in sandy soils of different factors such as moisture, bacteria, and so on (Othman et al., 2004; Moreno et al., 2007; Hanna et al., 2013). We found that specific bacteria were selectively enriched in developing *K. hirsuta* rhizosheath, which led to a significant switch in bacterial composition at different moisture and sterilization of soil. The data indicate that the choice of soil sterilization treatment (unsterilized soil vs. sterilized soil) rather than moisture level played a very critical role in the variation of the bacterial community.

Bacterial communities during rhizosheath formation in unsterilized soil varied more significantly than those in sterilized soil, a result that was mainly due to the enrichment of genera *Arthrobacter* and *Massilia* in unsterilization, as reflected by the high abundance of OTU16 (*Arthrobacter*) and OTU10 (*Massilia*). The genera *Massilia* mainly enrichment in sterilization, as reflected by the high abundance of OTU10 (*Massilia*). Several pieces of evidence indicate that the process of rhizosheath formation is responsible for the accumulation of specific bacteria from the rhizosphere. First, the physicochemical nature of soil can be altered and shaped by the specific rhizosphere community (Long et al., 2009). Second, specific bacterial communities are strongly influenced by soil moisture (Pereira et al., 2016). In this study, we observed a large variation in the bacterial composition of the rhizosheath under different conditions, which suggests that the specific bacterial community plays a key role in the establishment of rhizosheath formation.

*Arthrobacter* and *Massilia* are common bacteria in soil; in a previous study, species of *Arthrobacter* were isolated from the tomato rhizosphere and strains with high phosphate-solubilizing ability were then tested against a wide range of temperature, pH, and environmental stresses (Banerjee et al., 2010). Those strains also exhibited various plant growth-promoting and biocontrol activities, including indole acetic acid production, and have the potential to be used as plant growth-promoting rhizobacteria (Faure et al., 2013). In another study, a strain of *Massilia* significantly increased the growth of potato nodal explants in tissue culture (Turnbull et al., 2012); these reports indicate the existence of a molecular signal recognition system for the accumulation of specific groups of bacteria in the rhizosphere by plants.

Recruitment of microaggregates by the rhizosheath structure may also drive greater microbial diversity (Bach et al., 2018). In this study, *K. hirsuta* with a strong rhizosheath was more successfully established in unsterilized or sterilized soil. This result explains why rhizosheath weight is influenced by different moisture levels and why rhizosheath formation can be optimized to a particular moisture level.

### Growth-Promoting Activity of Special Microbial Underlying Rhizosheath Formation

Rhizosheath structure is a topic of interest in regard to the promotion of microbial diversity to enhance plant growth (e.g., nitrogen fixation) and protection under stress conditions (e.g., exopolysaccharide production) (Othman et al., 2004; Ashraf et al., 2006). The rhizosheath bacterial community is considered to be a potentially effective tool for plant growth (Sessitsch et al., 2012).

The abundance of *Arthrobacter*, *Massilia*, *Sphingomonas*, and *Cytophagaceae* was significantly increased in condition of unsterilization and sterilization, which have been successfully cultured and have been reported to promote the growth of several plant species (Zhang et al., 2019). In this study, a mixture of three strains of bacteria isolated from the *K. hirsuta* rhizosheath (OTU16, OTU6, and OTU10) significantly increased root weight. We also found that OTU16, OTU10, and a co-mixture significantly positively influenced the rhizosheath weight of *K. hirsuta.* OTU16 was found to play an important recruitment role in rhizosheath formation in unsterilized soil, while OTU10 exhibited a similar key role in sterilized soil. A mixture of strains, rather than a single strain, may have a stronger contribution to variation of the bacterial community to influence root and rhizosheath weights. Other studies have provided evidence that the composition of special microbe resources, namely, root exudates such as endogenous hormones, plays a critical role in the recruitment of specific bacteria from rhizosphere soil and the process of rhizosheath formation (Bais et al., 2006; Long et al., 2009). These results suggest that different strains have specific functions in *K. hirsuta* root growth and rhizosheath formation. Because our inoculation experiment used seedlings from only a single set of *K. hirsuta* plants, however, this hypothesis remains to be confirmed. In future studies, we accordingly plan to explore whether uniform effects are observed across various growth periods in additional plants including those from the same geographical source.

## Conclusion

Our data verified that rhizosheaths of *K. hirsuta* easily formed in relatively drier soil. Sterilized soil rather than moisture level played a very critical role in the variation of the bacterial community. *Arthrobacter* and *Massilia* play an important recruitment role in rhizosheath formation in unsterilized soil; *Massilia* mainly provides enrichment in sterilization, suggesting that the recognition mechanism may be conserved special bacteria in the *K. hirsuta* root growth and rhizosheath formation. The underlying physiological and molecular mechanisms need further elucidation. Taken together, our results provide a novel understanding of the physiological and molecular changes during the *K. hirsuta* rhizosheaths formation and environmental adaptation of sandy plants on the TP.

## Data Availability Statement

The data presented in the study are deposited in the NCBI (http://www.ncbi.nlm.nih.gov/bioproject/790485) repository, accession number PRJNA790485.

## Author Contributions

CC and YC contributed to the study design. CC, YC, QZ, JH, YL, and WL were involved in drafting the manuscript and agreed to be accountable for the work. All authors read and approved the final version of the manuscript.

## Conflict of Interest

The authors declare that the research was conducted in the absence of any commercial or financial relationships that could be construed as a potential conflict of interest.

## Publisher’s Note

All claims expressed in this article are solely those of the authors and do not necessarily represent those of their affiliated organizations, or those of the publisher, the editors and the reviewers. Any product that may be evaluated in this article, or claim that may be made by its manufacturer, is not guaranteed or endorsed by the publisher.
