# Supplementary material for: Specific Rhizobacteria Responsible in the Rhizosheath System of Kengyilia hirsuta
Source: Front Plant Sci. 2022 Jan 28;12:785971. doi: 10.3389/fpls.2021.785971 (PMC8832163; doi:10.3389/fpls.2021.785971)
Supplement: Supplementary file 1 [file Image_1.pdf]

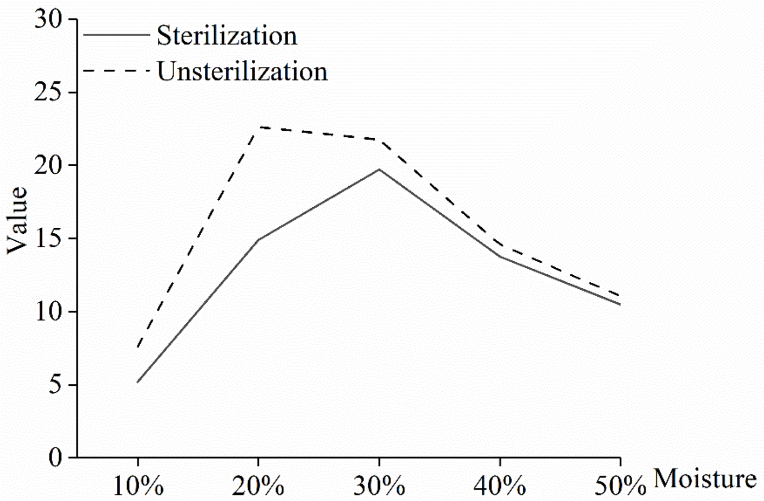

Supplementary Figure 1 The interaction between soil treatment and water difference in the process of rhizosheath weight
