# Supplementary figures and images for: Specific Rhizobacteria Responsible in the Rhizosheath System of Kengyilia hirsuta
Source: Front Plant Sci. 2022 Jan 28;12:785971. doi: 10.3389/fpls.2021.785971 (PMC8832163; doi:10.3389/fpls.2021.785971)

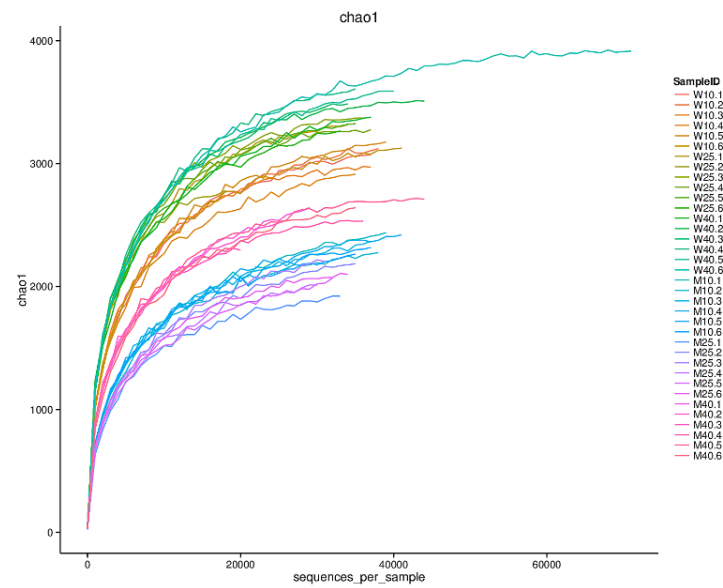

**Supplementary Figure 2 Rarefaction curve based on high-throughput sequencing.**

Supplement: Supplementary file 2 [file Image_2.pdf]
