# Supplementary material for: Specific Rhizobacteria Responsible in the Rhizosheath System of Kengyilia hirsuta
Source: Front Plant Sci. 2022 Jan 28;12:785971. doi: 10.3389/fpls.2021.785971 (PMC8832163; doi:10.3389/fpls.2021.785971)
Supplement: Supplementary file 3 [file Image_3.pdf]

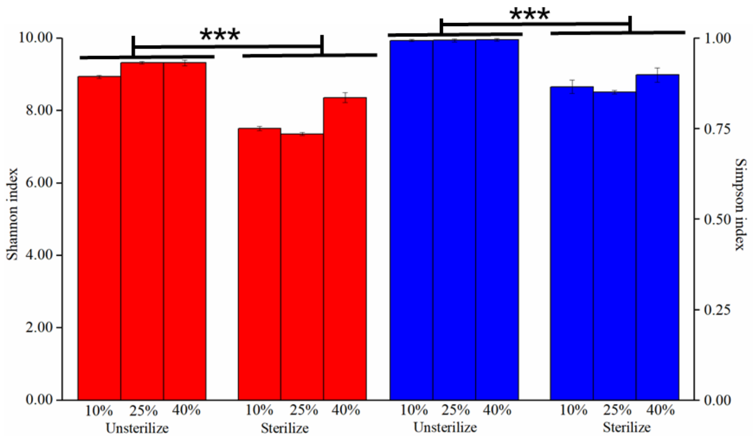

Supplementary Figure 3 Shannon index in Alpha diversity of Rhizosheath soil bacteria

***indicate significant differences at $P < 0.01$.
