# Supplementary material for: Specific Rhizobacteria Responsible in the Rhizosheath System of Kengyilia hirsuta
Source: Front Plant Sci. 2022 Jan 28;12:785971. doi: 10.3389/fpls.2021.785971 (PMC8832163; doi:10.3389/fpls.2021.785971)
Supplement: Supplementary file 4 [file Table_1.docx]

**Supplementary Table 1 Statistics on valid tags generated by high-throughput sequencing of *K. hirsuta* rhizosheath samples**

| **Sample_**  **ID** | **Valid_**  **tags** | **Valid min Length** | **Valid mean Length** | **Valid max Length** | **OTU_**  **counts** | **Total_**  **OTUs** | **Valid_**  **percent** |
| --- | --- | --- | --- | --- | --- | --- | --- |
| W10.1 | 37511 | 322 | 428.19 | 571 | 2482 | 6222 | 85.18% |
| W10.2 | 38751 | 304 | 428.15 | 571 | 2507 | 6222 | 84.65% |
| W10.3 | 37306 | 413 | 428.14 | 462 | 2462 | 6222 | 83.09% |
| W10.4 | 35390 | 356 | 428.03 | 571 | 2354 | 6222 | 85.48% |
| W10.5 | 39928 | 377 | 428.06 | 462 | 2537 | 6222 | 83.62% |
| W10.6 | 41043 | 304 | 428.27 | 462 | 2529 | 6222 | 86.09% |
| W25.1 | 34482 | 377 | 428.86 | 462 | 2696 | 6222 | 85.25% |
| W25.2 | 18818 | 322 | 428.57 | 462 | 2189 | 6222 | 86.70% |
| W25.3 | 36523 | 301 | 428.71 | 464 | 2799 | 6222 | 85.66% |
| W25.4 | 37847 | 413 | 428.87 | 464 | 2800 | 6222 | 85.95% |
| W25.5 | 35669 | 340 | 428.72 | 464 | 2730 | 6222 | 86.51% |
| W25.6 | 33094 | 411 | 428.33 | 458 | 2618 | 6222 | 84.32% |
| W40.1 | 37802 | 295 | 428.78 | 462 | 2722 | 6222 | 83.07% |
| W40.2 | 44698 | 377 | 429.06 | 455 | 2996 | 6222 | 84.57% |
| W40.3 | 34668 | 284 | 429.10 | 462 | 2795 | 6222 | 83.70% |
| W40.4 | 35173 | 329 | 428.91 | 455 | 2858 | 6222 | 84.39% |
| W40.5 | 40697 | 295 | 428.19 | 462 | 2909 | 6222 | 84.05% |
| W40.6 | 71468 | 327 | 428.6 | 462 | 3392 | 6222 | 83.80% |
| M10.1 | 39542 | 353 | 430.75 | 575 | 1830 | 6222 | 82.57% |
| M10.2 | 38692 | 377 | 430.82 | 575 | 1765 | 6222 | 83.32% |
| M10.3 | 37359 | 336 | 430.92 | 575 | 1807 | 6222 | 82.46% |
| M10.4 | 41693 | 413 | 431.16 | 571 | 1871 | 6222 | 81.72% |
| M10.5 | 37371 | 322 | 430.99 | 571 | 1801 | 6222 | 81.18% |
| M10.6 | 35077 | 319 | 431.00 | 575 | 1744 | 6222 | 81.24% |
| M25.1 | 33677 | 414 | 432.44 | 575 | 1513 | 6222 | 82.69% |
| M25.2 | 35394 | 413 | 432.21 | 575 | 1652 | 6222 | 80.44% |
| M25.3 | 31605 | 327 | 432.24 | 575 | 1613 | 6222 | 80.60% |
| M25.4 | 30740 | 327 | 432.25 | 571 | 1544 | 6222 | 82.59% |
| M25.5 | 32756 | 322 | 432.31 | 571 | 1532 | 6222 | 82.02% |
| M25.6 | 34383 | 413 | 432.2 | 575 | 1613 | 6222 | 80.73% |
| M40.1 | 29565 | 333 | 431.51 | 571 | 1984 | 6222 | 81.93% |
| M40.2 | 26518 | 336 | 431.59 | 572 | 1903 | 6222 | 82.72% |
| M40.3 | 20783 | 304 | 431.62 | 571 | 1755 | 6222 | 83.04% |
| M40.4 | 36644 | 377 | 431.75 | 571 | 2095 | 6222 | 82.66% |
| M40.5 | 44312 | 325 | 431.88 | 575 | 2249 | 6222 | 80.21% |
| M40.6 | 35883 | 345 | 431.28 | 575 | 2049 | 6222 | 83.62% |
