# Supplementary material for: Specific Rhizobacteria Responsible in the Rhizosheath System of Kengyilia hirsuta
Source: Front Plant Sci. 2022 Jan 28;12:785971. doi: 10.3389/fpls.2021.785971 (PMC8832163; doi:10.3389/fpls.2021.785971)
Supplement: Supplementary file 5 [file Table_2.docx]

**Supplementary Table 2 The abundance of rhizosphere soils in soil treatment and moisture treatment groups**

| **Soil treatment** | **PD_whole_tree** | **Chao1** | **Goods_coverage** | **Observed_species** |
| --- | --- | --- | --- | --- |
| Mann-Whitney U | 24 | 0.001 | 0.005 | 0.002 |
| Sig. | 0.003 | 0.002 | 0.00 | 0.001 |
| moisture |  | | | |
| chi-square | 10.5 | 5.059 | 5.209 | 4.108 |
| Sig. | 0.005 | 0.08 | 0.074 | 0.128 |
