# Supplementary material for: Specific Rhizobacteria Responsible in the Rhizosheath System of Kengyilia hirsuta
Source: Front Plant Sci. 2022 Jan 28;12:785971. doi: 10.3389/fpls.2021.785971 (PMC8832163; doi:10.3389/fpls.2021.785971)
Supplement: Supplementary file 6 [file Table_3.docx]

**Supplementary Table 3 The community differences between the unsterilization and sterilization soils at different phylogenetic levels**

| **Phy** | **Unsterilization** | | | | **Sterilization** | | | | |
| --- | --- | --- | --- | --- | --- | --- | --- | --- | --- |
| **Number** | **Taxonomy** | **10%** | **25%** | **40%** | **Taxonomy** | **10%** | **25%** | **40%** |  |
| 1 | Actinobacteria | 0.42208 | 0.39399 | 0.33845 | Proteobacteria | 0.48029 | 0.54972 | 0.47951 |  |
| 2 | Proteobacteria | 0.34662 | 0.31085 | 0.32684 | Actinobacteria | 0.24327 | 0.14364 | 0.15964 |  |
| 3 | Gemmatimonadetes | 0.11513 | 0.14385 | 0.16394 | Bacteroidetes | 0.09138 | 0.12485 | 0.14422 |  |
| 4 | Bacteroidetes | 0.05924 | 0.07017 | 0.08681 | Gemmatimonadetes | 0.10611 | 0.11490 | 0.11998 |  |
| 5 | Acidobacteria | 0.03144 | 0.04310 | 0.03973 | Firmicutes | 0.03919 | 0.03453 | 0.04315 |  |
| 6 | Firmicutes | 0.00713 | 0.00805 | 0.00918 | Acidobacteria | 0.01560 | 0.01643 | 0.02471 |  |
| 7 | Chloroflexi | 0.00687 | 0.00560 | 0.00506 | Fibrobacteres | 0.00644 | 0.00320 | 0.01090 |  |
| 8 | Fibrobacteres | 0.00039 | 0.00440 | 0.00841 | WCHB1_60 | 0.00638 | 0.00292 | 0.00327 |  |
| 9 | WCHB1_60 | 0.00206 | 0.00620 | 0.00431 | Cyanobacteria | 0.00525 | 0.00307 | 0.00228 |  |
| 10 | Nitrospirae | 0.00219 | 0.00451 | 0.00554 | Other | 0.00216 | 0.00232 | 0.00253 |  |
| 11 | Other | 0.00133 | 0.00231 | 0.00285 | Verrucomicrobia | 0.00093 | 0.00131 | 0.00211 |  |
| 12 | Elusimicrobia | 0.00044 | 0.00184 | 0.00378 | Chloroflexi | 0.00144 | 0.00093 | 0.00191 |  |
| 13 | Cyanobacteria | 0.00346 | 0.00108 | 0.00052 | Chlorobi | 0.00092 | 0.00106 | 0.00134 |  |
| 14 | Verrucomicrobia | 0.00039 | 0.00151 | 0.00128 | Elusimicrobia | 0.00011 | 0.00005 | 0.00177 |  |
| 15 | Saccharibacteria | 0.00043 | 0.00118 | 0.00079 | Gracilibacteria | 0.00000 | 0.00049 | 0.00050 |  |
| 16 | Chlorobi | 0.00050 | 0.00072 | 0.00091 | Armatimonadetes | 0.00023 | 0.00040 | 0.00032 |  |
| 17 | Parcubacteria | 0.00001 | 0.00029 | 0.00068 | Chlamydiae | 0.00006 | 0.00004 | 0.00067 |  |
| 18 | Gracilibacteria | 0.00000 | 0.00000 | 0.00035 | Saccharibacteria | 0.00009 | 0.00007 | 0.00037 |  |
| 19 | Armatimonadetes | 0.00013 | 0.00010 | 0.00011 | Parcubacteria | 0.00000 | 0.00000 | 0.00046 |  |
| 20 | TM6 | 0.00003 | 0.00009 | 0.00018 | Nitrospirae | 0.00013 | 0.00007 | 0.00026 |  |
| 21 | JL_ETNP_Z39 | 0.00007 | 0.00007 | 0.00012 | TM6 | 0.00000 | 0.00002 | 0.00003 |  |
| 22 | Planctomycetes | 0.00001 | 0.00003 | 0.00013 | JL_ETNP_Z39 | 0.00001 | 0.00000 | 0.00004 |  |
| 23 | Tenericutes | 0.00000 | 0.00002 | 0.00001 | Planctomycetes | 0.00000 | 0.00001 | 0.00003 |  |
| 24 | SM2F11 | 0.00000 | 0.00002 | 0.00001 | SM2F11 | 0.00000 | 0.00000 | 0.00001 |  |
| 25 | Deinococcus_Thermus | 0.00002 | 0.00000 | 0.00000 | Fusobacteria | 0.00000 | 0.00001 | 0.00000 |  |
| 26 | Chlamydiae | 0.00000 | 0.00000 | 0.00001 | Tenericutes | 0.00000 | 0.00000 | 0.00000 |  |
| 27 | Fusobacteria | 0.00000 | 0.00001 | 0.00000 | Deinococcus_Thermus | 0.00000 | 0.00000 | 0.00000 |  |
| **Class** | **Unsterilization** | | | | **Sterilization** | | | | |
| **Number** | **Taxonomy** | **10%** | **25%** | **40%** | **Taxonomy** | **10%** | **25%** | **40%** |  |
| 1 | Actinobacteria | 0.26928 | 0.23525 | 0.20546 | Betaproteobacteria | 0.28194 | 0.31972 | 0.20067 |  |
| 2 | Alphaproteobacteria | 0.18679 | 0.14570 | 0.16544 | Actinobacteria | 0.22223 | 0.12808 | 0.14084 |  |
| 3 | Gemmatimonadetes | 0.11513 | 0.14385 | 0.16394 | Alphaproteobacteria | 0.13467 | 0.15630 | 0.14390 |  |
| 4 | Thermoleophilia | 0.11074 | 0.09730 | 0.08153 | Gemmatimonadetes | 0.10611 | 0.11490 | 0.11998 |  |
| 5 | Betaproteobacteria | 0.08795 | 0.10198 | 0.09354 | Sphingobacteriia | 0.07318 | 0.10393 | 0.09471 |  |
| 6 | Sphingobacteriia | 0.05062 | 0.05212 | 0.05319 | Gammaproteobacteria | 0.03828 | 0.04778 | 0.08231 |  |
| 7 | Deltaproteobacteria | 0.02362 | 0.04718 | 0.04652 | Bacilli | 0.03886 | 0.03436 | 0.04287 |  |
| 8 | Acidimicrobiia | 0.03112 | 0.04614 | 0.03813 | Deltaproteobacteria | 0.02516 | 0.02479 | 0.04725 |  |
| 9 | Acidobacteria | 0.02375 | 0.03666 | 0.03410 | Cytophagia | 0.01675 | 0.02012 | 0.04599 |  |
| 10 | Gammaproteobacteria | 0.04799 | 0.01540 | 0.02047 | Acidobacteria | 0.01442 | 0.01545 | 0.02211 |  |
| 11 | Cytophagia | 0.00779 | 0.01774 | 0.03316 | Thermoleophilia | 0.01121 | 0.00658 | 0.00813 |  |
| 12 | Bacilli | 0.00650 | 0.00725 | 0.00770 | Acidimicrobiia | 0.00771 | 0.00728 | 0.00929 |  |
| 13 | Holophagae | 0.00768 | 0.00644 | 0.00562 | Fibrobacteria | 0.00644 | 0.00320 | 0.01090 |  |
| 14 | Fibrobacteria | 0.00039 | 0.00440 | 0.00841 | ML635J_21 | 0.00524 | 0.00305 | 0.00218 |  |
| 15 | Nitrospira | 0.00219 | 0.00451 | 0.00554 | Holophagae | 0.00118 | 0.00098 | 0.00259 |  |
| 16 | ML635J_21 | 0.00000 | 0.00000 | 0.00000 | Nitrospira | 0.00000 | 0.00000 | 0.00000 |  |
| **Genus** | **Unsterilization** | | | | **Sterilization** | | | | |
| **Number** | **Taxonomy** | **10%** | **25%** | **40%** | **Taxonomy** | **10%** | **25%** | **40%** |  |
| 1 | Gemmatimonas | 0.05062 | 0.05804 | 0.06798 | Massilia | 0.20921 | 0.25262 | 0.09957 |  |
| 2 | Arthrobacter | 0.05216 | 0.06466 | 0.04089 | Gemmatimonas | 0.06176 | 0.05969 | 0.06471 |  |
| 3 | Sphingomonas | 0.06422 | 0.03453 | 0.05663 | Sphingomonas | 0.03728 | 0.03359 | 0.04263 |  |
| 4 | Pseudonocardia | 0.03982 | 0.03970 | 0.04007 | Arthrobacter | 0.04863 | 0.03007 | 0.02891 |  |
| 5 | Patulibacter | 0.05076 | 0.01589 | 0.01594 | Flavisolibacter | 0.02154 | 0.03167 | 0.02067 |  |
| 6 | Nocardioides | 0.03282 | 0.01013 | 0.00935 | Bacillus | 0.02583 | 0.01719 | 0.02725 |  |
| 7 | Solirubrobacter | 0.01132 | 0.01525 | 0.01298 | Lysobacter | 0.02022 | 0.01146 | 0.03663 |  |
| 8 | Bryobacter | 0.01098 | 0.01240 | 0.01241 | Nocardioides | 0.02807 | 0.03045 | 0.00633 |  |
| 9 | Massilia | 0.01430 | 0.01020 | 0.00982 | Niastella | 0.01297 | 0.01666 | 0.02158 |  |
| 10 | Haliangium | 0.00437 | 0.01455 | 0.01461 | Phenylobacterium | 0.01146 | 0.02286 | 0.01187 |  |
| 11 | Flavisolibacter | 0.01231 | 0.01022 | 0.01061 | Micromonospora | 0.02040 | 0.00686 | 0.01351 |  |
| 12 | Segetibacter | 0.01166 | 0.01004 | 0.01068 | Ramlibacter | 0.01273 | 0.00788 | 0.01834 |  |
| 13 | Gaiella | 0.00877 | 0.01153 | 0.00971 | Streptomyces | 0.02746 | 0.00541 | 0.00510 |  |
| 14 | Noviherbaspirillum | 0.01095 | 0.00986 | 0.00892 | Bryobacter | 0.01088 | 0.01240 | 0.01372 |  |
| 15 | Candidatus_Solibacter | 0.00669 | 0.01088 | 0.00936 | Segetibacter | 0.01233 | 0.01007 | 0.00754 |  |
| 16 | Bacillus | 0.00000 | 0.00000 | 0.00000 | Pseudonocardia | 0.00000 | 0.00000 | 0.00000 |  |
| 17 | Lysobacter | 0.00000 | 0.00000 | 0.00000 | Patulibacter | 0.00000 | 0.00000 | 0.00000 |  |
| 18 | Niastella | 0.00000 | 0.00000 | 0.00000 | Solirubrobacter | 0.00000 | 0.00000 | 0.00000 |  |
| 19 | Phenylobacterium | 0.00000 | 0.00000 | 0.00000 | Haliangium | 0.00000 | 0.00000 | 0.00000 |  |
| 20 | Micromonospora | 0.00000 | 0.00000 | 0.00000 | Gaiella | 0.00000 | 0.00000 | 0.00000 |  |
| 21 | Ramlibacter | 0.00000 | 0.00000 | 0.00000 | Noviherbaspirillum | 0.00000 | 0.00000 | 0.00000 |  |
| 22 | Streptomyces | 0.00000 | 0.00000 | 0.00000 | Candidatus_Solibacter | 0.00000 | 0.00000 | 0.00000 |  |
